# Special Issue: Structure, Function and Evolution of Protein Domains

**DOI:** 10.3390/ijms23116201

**Published:** 2022-05-31

**Authors:** Sailen Barik

**Affiliations:** EonBio, 3780 Pelham Drive, Mobile, AL 36619, USA; eonbiohelp@gmail.com

Essentially, all proteins perform their biological roles through the use of specific domains that number in the hundreds, if not thousands. The domains are often recognized by their consensus sequences, various motifs, higher order of structural features, and by structure–function analyses. This Special Issue, “Structure, Function and Evolution of Protein Domains”, presents a collection of ten articles (six original research articles and four reviews) from distinguished experts, covering various aspects of this important area of molecular biology.

Adameyko et al. [1] performed detailed research on the ferritin family of sponges (*Porifera*), the oldest Metazoa that exhibit unique morphological plasticity and reversible cellular aggregation. Ferritins constitute a conservative family of proteins present ubiquitously in all species and that play important roles in immune response, redox stress, and cellular differentiation. The authors used a comprehensive suite of proteomics, spectral microscopy, and bioinformatics, and found duplicated conservative HdF1a/b and atypical HdF2 genes, coding for ferritin, in two cold-water sponges (*Halisarca dujardini* and *Halichondria panicea*). Expression analyses revealed multiple transcripts and differential regulation during sponge dissociation/reaggregation. Moreover, the presence of the MRE and HRE motifs in the promotor regions of the conserved ferritin genes and the iron-responsive element (IRE) motif in the mRNAs indicated that their expression is regulated by cellular iron and oxygen levels. Specific staining of protein gels and mass spectrometry provided direct evidence of the presence of ferric ions and ferritins in multi-subunit complexes. In silico 3D modeling also revealed the iron-binding capacity of HdF1 and HpF1 at the ferroxidase center, and its absence in atypical HdF2. Remarkably, the authors found atypical ferritin sequences that lack the typical IRE motif in many invertebrate genomes, which are, therefore, unlikely to be regulated by iron. The role of these atypical iron non-responsive ferritins should be the subject of exciting future research.

Barik [2] offered a detailed analytical and comparative review of a special kind of protein motif, found as repeats that are commonly 34- and 35-amino acid residues long, and are known, respectively, as tetratricopeptide (TPRs) and pentatricopeptide repeats (PPRs). Both repeats are common in natural proteins, PPRs being particularly abundant in plants. In both classes, each repeat unit forms an antiparallel bihelical structure, so that several such units in a polypeptide are arranged in a parallel fashion. Barik essentially collected all these repeats that actually occur in nature and found that specific amino acid residues, namely Gly, Pro, Ala and Trp, were placed at distinct locations in higher order structures. While most TPRs occurred in repeats of three, PPRs exhibited a much greater latitude in repeat numbers, ranging from 1 to 30 or more, separated by linkers of diverse sequences and lengths. Interestingly, notwithstanding their “repeat” designation, the majority of PPR units were in single patches, and the longer multi-PPR tandems occurred in a decreasing order. The multi-PPR domains formed superhelical vortices that were similar to those of TPR and appeared to be governed by interhelical angles and the energy of side-chain interactions rather than the spacers. Barik’s findings should be useful in understanding the role and evolution of the PPR domains and creating designer PPRs for targeted protein–protein and protein–ligand interactions.

Epigenetic remodeling of the eukaryotic chromatin is fundamental to the expression of genetic information and regulates a multitude of biological processes, such as DNA replication and repair, apoptosis, chromosome segregation, development, and pluripotency. Conversely, abnormalities of chromatin remodeling proteins are associated with genetic diseases, including cancer. Remodeling is largely regulated by histone modification, controlled by individual proteins and protein complex subunits that recognize different modifications of N-terminal histone tails. In this Special Issue, Chugunov et al. [3] focused on the structure and evolution of the double PHD finger (DPF) domain of PHF10, the specific subunit of the chromatin remodeling complex, PBAF. The plant homeodomain fingers (PHDs) are zinc-finger-containing domains that recognize modified N-terminal tails of H3 histones, and thus, play a cardinal role in remodeling by PBAF. As the authors elegantly summarized, PHF10 is a unique protein, expressed as a total of four isoforms, two with the DPF domain and two without. These isoforms are all subunits of the PBAF chromatin remodeling complex and determine its nucleosome-binding specificity. Homology modeling suggests that the PHF10 DPF domain has unique conserved structural features that enable specific isoforms to bind the H3K14ac, H3R2, and H4K16ac markers of actively transcribed chromatin, but not the functionally similar marker H3K4me3. The latter may use PBAF complexes with subunits other than PHF10. The authors raise provocative scenarios of evolution of the various PHF10 subunits through the phylogenetic tree and how they may relate to the composition and specificity of the PBAF holocomplex. Such knowledge may allow targeting chromatin remodeling as a major therapeutic strategy in genetic abnormalities and in the treatment of several cancers.

Lack of efficient biophysical techniques is a major bottleneck in monitoring real-time protein dynamics. Fujimura et al. [4] contributed a masterly review of the diffracted X-ray tracking (DXT) method, an X-ray-based single-molecule technique that monitors the internal motion of biomolecules in an aqueous solution. It analyzes the trajectories of Laue spots generated from the attached gold nanocrystals. High-intensity X-rays from synchrotron radiation enable measurements with microsecond timescale and picometer spatial scale that provides high-resolution intramolecular information. DXT has been applied to various membrane proteins due to its superior spatiotemporal resolution. The authors described the basic principles of DXT with experimental details, as well as its recent and extended applications to membrane proteins and in living cells, using highly illustrative and reader-friendly graphics. They also suggested directions of future development and applications of the technique.

From the earliest days of molecular biology, studies of prokaryotes have contributed fundamental knowledge to our understanding of the structure and function of proteins and genes. In continuing the tradition, Gala and coworkers [5] report on the nature of allosteric inter-domain contacts in the bacterial heat shock protein 70 (Hsp70) and offer an interesting glimpse into the constraints of structural evolution. Specifically, by analyzing gene sequence variations with the higher order structures of Hsp70, they discovered that these amino acid contacts are located in regions that avoid chromosomal insertion and deletion events (indels). This is because the indels can be highly disruptive for the mechanical events required to orchestrate proper pairing interactions at communicating interfaces.

Lorenz et al. [6] drew our attention to eukaryotic transcription factors, specifically a protein-protein interaction domain known as Krüppel-associated box (KRAB), commonly found together with the Cys2His2 (C2H2) Zn-finger domain. This subclass, referred as KRAB Zn-finger (KZNF) proteins, first appeared ~400 million years ago and is widely and abundantly distributed in all extant species from invertebrates to mammals; humans, for example, possess roughly 400 KZNF genes. By stratifying the ancestral KRAB sequences with the aid of a hidden Markov model and comparing them with the human homologs, Lorenz et al. identified the amino acid changes that transformed an inactive ancestral-like domain into a functional modern domain, assayed by complexing with TRIM28 that resulted in transcriptional silencing. This was followed by in-depth structural analysis that provided important insights into the interaction of the KRAB domain side-chains with TRIM28 and the transition of this interaction during evolution. These studies provide a road map for understanding the evolution of structure–function relationship in the biological world.

The importance of Zn-fingers in transcription-regulatory proteins is further underscored by another study, in which Tikhonova et al. [7] explored the dimerization activity of a disordered N-terminal domain in the CLAMP protein of *Drosophila melanogaster* (the fruit fly). CLAMP is an essential C2H2 Zn-finger protein that binds to thousands of sites in the *Drosophila* chromosome and is involved in multiple functions, including X chromosome dosage compensation, embryonic reprogramming, and chromatin remodeling, reminiscent of the DPF domain of PHF10, described by Chugunov et al. [3]. Whereas Zn-fingers generally bind DNA, the proteins containing Zn-fingers possess other domain(s) that dictate protein–protein interactions, as also mentioned by Lorenz et al. [6]. Likewise, the CLAMP proteins contain a conserved N-terminal homodimerization domain that facilitates long distance interaction between chromosomal loci. Tikhonova et al. used biophysical techniques, including nuclear magnetic resonance (NMR) and small-angle X-ray scattering (SAXS) to demonstrate that this domain lacks secondary structure and has features of intrinsically disordered regions (IDR), even though structure prediction algorithms suggested the presence of beta-sheets. Functional analyses showed that the domain is essential for CLAMP functions in vivo and that its deletion results in lethality. These results established CLAMP as only the second architectural Zn-finger protein with an IDR, making it worthy of further analysis.

Villalobos et al. [8] reviewed another transcription factor, namely human FoxP, and showed that it can serve as a tractable model for the evolution and function of three-dimensional (3D) domain swapping (DS), a common theme in the formation of multimeric protein complexes. Biophysical studies on FoxP have suggested that the dynamics of the polypeptide chain are crucial to decrease the energy barrier of 3D-DS, enabling fast oligomerization. Comparison of biophysical parameters between human FoxP proteins suggested differential evolutionary strategies that favored homoassociation and presaged the likelihood of heteroassociations, which was directly relevant to their transcription regulatory function. The authors also point out that the FoxP proteins encode several accessory domains, such as ZFD and LZD, which have been extensively studied in relevance to their roles in DNA-binding and dimerization, suggesting a functional redundancy or synergy between them. Clearly, the analysis of the structural elements of the FoxP proteins and their functional implication deserve extensive investigations.

Intrinsically disordered regions (IDRs), as exemplified in Tikhonova et al. [7], play a cardinal role in the structural and functional dynamics of many proteins and have continued to receive increasing attention. In another research article in this Special Issue, Wang et al. [9] identified and investigated the distinctive properties of >22,000 consecutively disordered regions (CDRs) that lie either inside or outside of protein domains, and explored their functional significance. The authors conducted a comprehensive analysis of the various differences between these two types of CDRs that led to several fascinating distinctions in physical properties, stability, post-translational modification, abundance, and evolutionary rate. These results provide valuable new insights into consecutively disordered regions in naturally occurring proteins and their structural and functional implications.

Lastly, Yu et al. [10] recognized the profound potential of immune therapy, such as its emerging success in the treatment of cancers. They concentrated on the immune checkpoint protein B and T Lymphocyte Attenuator (BTLA) and its interacting protein, namely the herpes virus entry mediator (HVEM), as potential targets for drug development. The co-crystal structure of BTLA/HVEM revealed that the HVEM (26–38) fragment is the core sequence, directly involved on the interface. The authors, therefore, conducted a virtual evolution analysis of this sequence using saturation mutagenesis in silico, and selected mutants with lower binding energy, followed by confirmation of their higher affinity for BTLA in wet-lab experiments. They also defined the mechanism of the effects of the mutations by computational analysis. These results illustrate how viruses may mutate to regulate the host immune response. Additionally, the mutated peptide reported in this article can be a potent inhibitor to block BTLA/HVEM interaction, which establishes checkpoint pair inhibition as a major drug discovery regimen.
